# The effects of NLRP3 and MAVS gene polymorphisms on the risk of asthma: A case–control study

**DOI:** 10.1097/MD.0000000000032385

**Published:** 2022-12-23

**Authors:** Cai Xulong, Zhou Li, Yin Tongjin

**Affiliations:** a Department of Pediatrics, Yancheng Third People’s Hospital (The Sixth Affiliated Hospital of Nantong University), Yancheng, China.

**Keywords:** asthma, genes interaction, MAVS, NLRP3, polymorphisms

## Abstract

Genetic factors are important risk factors for asthma. Nucleotide-binding oligomerization domain-like receptor containing pyrin domain 3 (NLRP3) is closely associated with asthma. Mitochondrial antiviral signaling protein (MAVS) mediates the recruitment of NLRP3 to the mitochondria and activation of the NLRP3 inflammasome. The purpose of this study was to analyze the effects of NLRP3 and MAVS polymorphisms on the risk of asthma and the interactions between them. Children with asthma (n = 127) and healthy children (n = 100) were recruited between August, 2020 and July, 2021. Multiplex polymerase chain reaction and sequencing was used to analyze genotypes of single nucleotide polymorphisms. The multifactor dimensionality reduction statistical method was used to detect and model epistasis of gene–gene interactions. There were significant differences in the distribution of MVAS rs6515831 and NLRP3 rs10925023 genotypes between the asthma and healthy groups. Compared with rs6515831 TT genotype, the results showed that rs6515831CT genotype increased the risk of asthma (odds ratio: 2.243, 95% CI: 1.221–4.122, *P* = .009). Compared with rs10925023 GG genotype, the results showed that the risk of asthma in the population with rs10925023 TT genotype was lower (odd ratio: 0.643, 95% CI: 0.423–0.979, *P* = .039). In the genotype of the NLRP3 rs12048215 locus, the IgE level of asthma patients with genotype AG was lower than that of patients with genotype AA. The dendrogram model showed the strongest interaction between rs7272495 and rs10925023, which was expressed in a synergistic manner. Haplotype analysis revealed that rs10925023T/rs7272495G and rs10925023T/rs3272495A were statistically different in distribution between the two groups. The MAVS rs6515831 and NLRP3 rs10925023 polymorphisms were associated with the risk of asthma in children. There may be interactions between NLRP3 and MAVS polymorphisms in the risk of asthma.

## 1. Introduction

Asthma is a common chronic respiratory disease worldwide, which increases the burden on families and society. Chronic airway inflammation is the basic pathological feature of asthma. At present, asthma is believed to be a heterogeneous disease that is affected by the environment and heredity. Asthma attacks often result in coughing, wheezing, shortness of breath, and chest tightness.^[1]^ Common triggers for asthma include air pollution, allergens, viral respiratory infections, weather changes and exercise.^[2–6]^ The cells involve airway inflammation include *T* helper cells, lymphocytes, eosinophils, mast cells, basophils, macrophages, epithelial cells, dendritic cells, goblet cell, fibroblasts, smooth muscle cells, neuronal cells and endothelial cells.^[7,8]^

Nucleotide-binding oligomerization domain-like receptors (NLRs) are a group of pattern recognition receptors. The NLRs family has 22 members in humans and 34 in mice.^[9]^ The expression of NLRs in the cell nucleus and cytoplasm plays an important role in inflammation and immune response.^[10,11]^ Nucleotide-binding oligomerization domain-like receptor containing pyrin domain 3 (NLRP3) is a member of the NLRs family. A study indicated that NLRP3 plays a key role in helper *T* cell (Th) 2 differentiation.^[12]^ Further study had revealed that NLRP3 was involved in the inflammatory response of Th2 and Th17 in asthmatic mice by inducing the expression and secretion of high mobility protein 1.^[13]^ Current studies suggested that pathogens and allergens could activate NLRP3.^[14]^ Activated NLRP3 recruits PYD and CARD domain containing and Caspase-1 to form a protein complex called NLRP3 inflammasome.^[15]^ The activation of NLRP3 inflammasome leads to the production of interleukin 1 (IL-1), which plays a key role in the induction of Th2 allergic inflammatory response.^[16–18]^ A previous study showed that NLRP3 participated in ovalbumin-mediated allergic airway inflammation independently of inflammasome.^[19]^ Studies have shown that the expression of the NLRP3 inflammasome is upregulated in the neutrophilic asthma phenotype.^[20,21]^ Thus, NLRP3 is closely associated with asthma.

The mitochondrial outer membrane is a platform for signal transduction of the mitochondrial antiviral signaling protein (MAVS) and the NLRP3 inflammasome.^[22]^ As a mitochondrial related adaptor molecule, MAVS mediates the recruitment of the NLRP3 to mitochondria, activation of NLRP3 inflammasome, and promotion of IL-1β production.^[23]^ This study analyzed the effects of NLRP3 and MAVS polymorphisms on the risk of asthma. Moreover, the interaction between the 2 genes was explored using multifactor dimensionality reduction (MDR).

## 2. Materials and Methods

### 2.1. Study subjects

This study was designed as a case–control study. Children with asthma and healthy children were recruited between August 2020 and July 2021. The children were aged between 6 and 14 years. The diagnostic basis for asthma was the Global Initiative for Asthma.^[24]^ Asthma was diagnosed according to the following criteria: cough, wheezing, shortness of breath, chest tightness, and variable expiratory airflow limitation. Healthy children without an allergic history, asthma history or immune disease were included. The study was approved by the ethics committee of the Yancheng Third People’s Hospital and informed consent was obtained from the guardians of the study subjects.

### 2.2. DNA extraction and genotyping

The peripheral blood 2 mL of the subjects were collected and stored at –80°C. DNA extraction shall be conducted according to the instructions of the Human Blood DNA Extraction Kit [Sangon Biotech (Shanghai) Co., Ltd.]. The extracted DNA samples were stored in a refrigerator at –80°C.

Multiplex polymerase chain reaction (PCR) and sequencing was used to analyze genotypes of single nucleotide polymorphisms (SNPs). A panel containing 5 target SNP sites (rs6515831 T > C, rs6084497 C > T, rs7272495 G > A, rs10925023 G > T and rs12048215 A > G) was designed. Library preparation was performed by 2 step PCR. The first round PCR reaction was set up as follows: DNA (10 ng/μL) 2 μL; amplicon PCR forward primer mix (10 μM) 1 μL; amplicon PCR reverse primer mix (10 μM) 1 μL; 2× PCR Ready Mix 15 μL (total 25 μL) (Kapa HiFi Ready Mix). The plate was sealed and PCR was performed in a thermal instrument (BIO-RAD, T100TM) using the following program: 1 cycle of denaturing at 98°C for 5 minutes, first 8 cycles of denaturation at 98°C for 30 seconds, annealing at 50°C for 30 seconds, elongation at 72°C for 30 seconds, followed by 25 cycles of denaturation at 98°C for 30 seconds, annealing at 66°C for 30 seconds, elongation at 72°C for 30 seconds and a final extension at 72°C for 5 minutes. Finally hold at 4°C. The PCR products were checked using electrophoresis in 1% (w/v) agarose gels in TBE buffer (Tris, boric acid, EDTA) stained with ethidium bromide (EB) and visualized under UV light. AMPure XP beads were used to purify the amplicon product. Next, the second round PCR was performed. The PCR reaction was set up as follows: DNA (10 ng/μL) 2 μL; universal P7 primer with barcode (10 μM) 1 μL; universal P5 primer (10 μM) 1 μL; 2× PCR Ready Mix 15 μL (total 30 μL) (Kapa HiFi Ready Mix). The plate was sealed and PCR was performed in a thermal instrument (BIO-RAD, T100TM) using the following program: 1 cycle of denaturing at 98°C for 3 minutes, then 5 cycles of denaturing at 94°C for 30 seconds, annealing at 55°C for 20 seconds, elongation at 72°C for 30 seconds, and a final extension at 72°C for 5 minutes. Then we used AMPure XP beads to purify the amplicon product. The libraries were then quantified and pooled. Paired-end sequencing of the library was performed on the HiSeq XTen sequencers (Illumina, San Diego, CA).

### 2.3. Multifactor dimensionality reduction statistical method for gene–gene interaction

MDR statistical method is a nonparametric statistical tool for detecting and modeling epistasis of gene–gene interactions.^[25]^ Based on the information theory, the model is displayed using dendrogram and circle graph. All interactions between SNPs show a percentage of entropy risk. A positive percentage score was designated as a synergistic interaction, whereas a score of 0 or less was designated as redundant or antagonistic.

### 2.4. Statistical analysis

The mean ± standard deviation or interquartile range was used to describe the characteristics of continuous variables. Student’s *t*-test or Mann–Whitney *U* test was used to analyze the differences in continuous variables between the two groups. The frequency and percentage were used to represent the characteristics of the counting data. The *χ*^2^-test was used to analyze differences in genotype and allele distribution between the two groups. Statistical analyses were performed using SPSS 22.0 (IBM Corp., Armonk, NY). And *P* < .05, the difference was statistically significant.

## 3. Results

### 3.1. Basic data characteristics of subjects

There were no significant differences in the distribution of age and sex between the two groups (Table 1). Total serum IgE levels were significantly higher in the asthma group than in the healthy. The proportion of eosinophils in children with asthma was higher than that in healthy children. There was a high proportion of allergic diseases and a history of allergies children with asthma.

**Table 1:** General information of subjects.

Characteristic	Asthma	Control	*P*
Age (yr, Mean ± SD)	8.016 ± 1.345	7.990 ± 1.605	.896
Sex (F/M)	49/78	44/56	.410
IgE (IU/mL, IQR)	352.4 (193.9, 774.5)	62.0 (43.1, 76.5)	<.001
Eosinophils (%)	4.1 ± 3.2	1.6 ± 0.8	<.001
FEV1/FVC (%)	75.4 ± 5.0	(–)	
Allergic diseases, n (%)	63 (49.6%)	0	
Allergic history, n (%)	82 (64.6%)	0	
Family history of asthma, n (%)	26 (20.5%)	0	

IQR = quartile range, SD = standard deviation.

### 3.2. Characteristics of MAVS and NLRP3 gene polymorphisms

SNPs in the human MAVS and NLRP3 genes with minor allele frequencies >20% were selected from the HapMap China database. Then the tag SNPs were screened using haploview 4.2 software (Broad Institute, Massachusetts) Three loci of MVAS (rs6515831, rs6084497 and rs7272495) and 2 loci of NLRP3 (rs10925023 and rs12048215) were selected and included in this study (Table 2). PCR primers for polymorphic loci were designed using online primer 3.0 software (http://primer3.ut.ee/).

**Table 2:** Primer for SNPs.

SNP	Allele		引物
rs6515831	T > C	Forward	5′-GAGAAAGCCAAGGGGCAGAT-3′
		Reverse	5′-TCTGTCAGGGCCTAGTGGAA-3′
rs6084497	C > T	Forward	5′-GCTGTGTTCTGCACGCTAGT-3′
		Reverse	5′-GTTACTTTTCTGCCGCCCCC-3′
rs7272495	G > A	Forward	5′-TTCAAGTGGTTGGGGACGTT-3′
		Reverse	5′-TCCCTTTGCCACCCCTAATG-3′
rs10925023	G > T	Forward	5′-CTCGATGTTGTATGCAGCCC-3′
		Reverse	5′-AGCAACACACAATGGCTTCC-3′
rs12048215	A > G	Forward	5′-GGCAGGGTATTGCAGGATGA-3′
		Reverse	5′-GGGTTTCTTGAATGTGCCATAA-3′

SNP = single nucleotide polymorphism.

Rs6515831, rs6084497, rs7272495, rs10925023 and rs12048215 were genotyped (Table 3). The distribution frequencies of the genotypes in the two groups were statistically analyzed. The MVAS rs6515831 genotypes were TT, CT and CC. There were significant differences in the distribution of MVAS rs6515831 genotypes between the asthma and healthy groups. Compared with rs6515831 TT genotype, the results showed that the risk of asthma in the population with rs6515831 CT genotype was higher (odds ratio: 2.243, 95% CI: 1.221–4.122, *P* = .009). The genotypes of NLRP3 rs10925023 were GG, GT and TT. There was a statistically significant difference in the distribution frequency of NLRP3 rs10925023 genotypes between the two groups. Compared with rs10925023 GG genotype, the results showed that the risk of asthma in the population with rs10925023 TT genotype was lower (odds ratio: 0.643, 95% CI: 0.423–0.979, *P* = .039). There were no significant differences in the distribution frequencies of the other SNPs between the two groups.

**Table 3:** Distribution characteristics of genotype in asthma group and healthy group.*

Gene	SNP		Genotype		*P*	Genotype vs genotype	OR (95% CI)	*P*
			Asthma	Control				
MAVS	rs6515831	TT	73 (57.5%)	74 (74.0%)	.018			
		CT	47 (37.0%)	22 (22.0%)		CT vs TT	2.243 (1.221–4.122)	.009
		CC	7 (5.5%)	4 (4.0%)		CC vs TT	1.345 (0.708–2.555)	.365
	rs6084497	CC	58 (45.7%)	45 (45.0%)	.406			
		CT	52 (40.9%)	46 (46.0%)		CT vs CC	0.865 (0.495–1.511)	.61
		TT	17 (13.4%)	9 (9.0%)		TT vs CC	1.173 (0.743–1.850)	.493
	rs7272495	AA	16 (12.6%)	18 (18.0%)	.306			
		AG	41 (32.3%)	32 (32.0%)		AG vs AA	0.918 (0.509–1.654)	.775
		GG	70 (55.1%)	50 (50.0%)		GG vs AA	0.810 (0.551–1.192)	.285
NLRP3	rs10925023	GG	48 (37.8%)	27 (27.0%)	.033			
		GT	64 (50.4%)	53 (53.0%)		GT vs GG	0.657 (0.358–1.206)	.175
		TT	15 (11.8%)	20 (20.0%)		TT vs GG	0.643 (0.423-0.979)	.039
	rs12048215	AA	63 (49.6%)	54 (54.0%)	.731			
		AG	56 (44.1%)	38 (38.0%)		AG vs AA	1.268 (0.725–2.218)	.405
		GG	8 (6.3%)	8 (8.0%)		GG vs AA	1.058 (0.615–1.818)	.839

MAVS = mitochondrial antiviral signaling protein, NLRP3 = Nucleotide-binding oligomerization domain-like receptor containing pyrin domain 3, OR = odds ratio, SNP = single nucleotide polymorphism.

* Adjusted for age, sex.

The relationship between genotype distribution and IgE level was analyzed (Table 4). The serum IgE level in asthmatic children with the rs12048215 AG genotype was lower than that in asthmatic children with the rs12048215 AA genotype (*P* = .018). No statistical difference was found in the distribution of genotype in different age groups.

**Table 4:** Analysis of genotype distribution according to clinical phenotype.

Gene	SNP	Genotype	N	IgE (IU/mL, IQR)	*P*	Genotype distribution [n (%)]*		*P*
						6–8 yr	≥9 yr	
MAVS	rs6515831	TT	73	374.1 (271.0, 796.0)	Ref.	49 (54.4)	24 (64.9)	.537
		CT	47	323.9 (132.3, 772.2)	.228	36 (40.0)	11 (29.7)	
		CC	7	373.4 (337.0, 881.7)	.845	5 (5.6)	2 (5.4)	
	rs6084497	CC	58	349.6 (199.8, 779.9)	Ref.	40 (44.4)	18 (48.6)	.894
		CT	52	362.9 (146.8, 792.2)	.945	38 (42.2)	14 (37.8)	
		TT	17	367.6 (303.7, 749.8)	.800	12 (13.3)	5 (13.5)	
	rs7272495	GG	70	369.7 (272.6, 772.6)	Ref.	51 (56.7)	19 (51.4)	.860
		AG	41	307.7 (139.4, 750.2)	.216	28 (31.1)	13 (35.1)	
		AA	16	453.4 (255.8, 877.8)	.594	11 (12.2)	5 (13.5)	
NLRP3	rs10925023	GG	48	324.3 (146.8, 696.9)	Ref.	34 (37.8)	14 (37.8)	.689
		GT	64	406.0 (186.6, 798.3)	.194	44 (48.9)	20 (54.1)	
		TT	15	547.4 (337.0, 858.9)	.081	12 (13.3)	3 (8.1)	
	rs12048215	AA	63	547.5 (206.1, 881.0)	Ref.	46 (51.1)	17 (45.9)	.100
		AG	56	332.1 (146.8, 593.3)	.018	36 (40.0)	20 (54.1)	
		GG	8	348.2 (147.1, 697.1)	.383	8 (8.9)	0 (0)	

IQR = quartile range, MAVS = mitochondrial antiviral signaling protein, NLRP3 = nucleotide-binding oligomerization domain-like receptor containing pyrin domain 3, OR = odds ratio, SNP = single nucleotide polymorphism.

* Percentages may not total 100 because of rounding.

### 3.3. MDR model analysis

**Figure 1.**
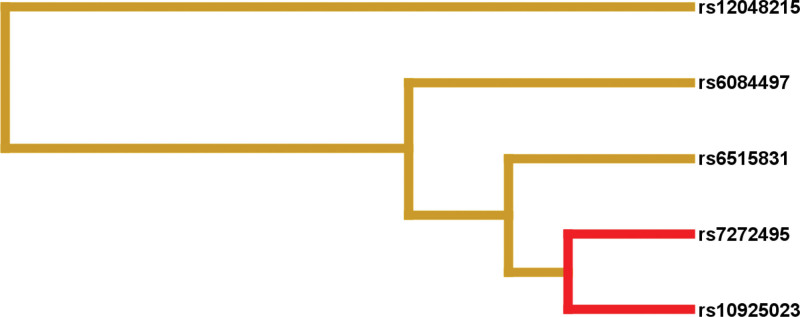
The dendrogram model of gene–gene interaction for asthma candidate genes. A dendrogram of the interactions showed SNPs that interacted synergistically or antagonistically. There was the strongest interaction between NLRP3 rs10925023 and MVAS rs7272495.

**Figure 2.**
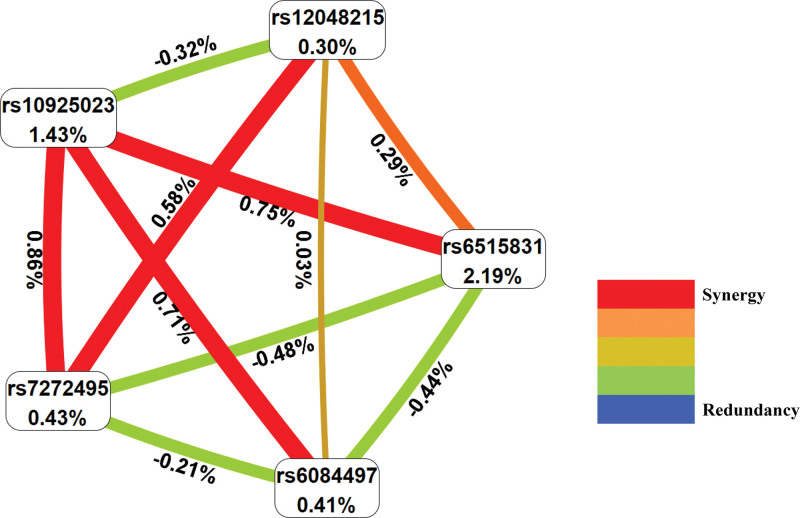
The circle graph model of gene–gene interaction for asthma candidate genes. The circular graph of interaction shown the gain synergy or redundancy percentage entropy. Red represents a high degree of synergy, orange represents a low degree, and gold represents the middle between synergy and redundancy. At the redundant end of the spectrum, the highest order is represented by blue and the lower order is represented by green.

Five SNPs were included in MDR analysis to assess their predictive potential and interactions. The dendrogram model showed how these 5 gene loci interacted with each other. The strongest interaction was observed between NLRP3 rs10925023 and MVAS rs7272495, which was expressed in a synergistic manner (Fig. 1). We analyzed the association between the rs10925023/rs7272495 haplotypes and the risk of asthma.^[26]^ Several possible haplotypes are listed in Table 5. By haplotype analyses, the haplotype TG (rs10925023/rs7272495) and TA (rs10925023/rs7272495) were statistically significant between the asthma and healthy groups. Circle graph model of information scores showed that MAVS rs6515831 percentage score (2.19%) was the most important of the 5 loci in the study (Fig. 2).

**Table 5:** The distribution of NLRP3 rs10925023/MAVS rs7272495 haplotypes frequencies between asthma group and control group [n (%)].

rs10925023/rs7272495 haplotypes	Case (n = 254)	Control (n = 200)	OR (95% CI)	*P*
G-G	116 (45.7)	69 (34.5)	Ref.	
T-G	65 (25.6)	62 (31.0)	0.624 (0.394–0.986)	.043
G-A	45 (17.7)	38 (19.0)	0.704 (0.417–1.190)	.190
T-A	28 (11.0)	31 (15.5)	0.537 (0.297–0.971)	.038

MAVS = mitochondrial antiviral signaling protein, NLRP3 = nucleotide-binding oligomerization domain-like receptor containing pyrin domain 3, OR = odds ratio.

## 4. Discussion

This study analyzed the risk of asthma from the perspective of genetic polymorphisms. Interestingly, some interesting results were obtained. The MAVS rs6515831 polymorphism was associated with asthma. Compared with the wild-type homozygous rs6515831 TT genotype, the results showed that the rs6515831 CT genotype increased the risk of asthma. At present, no other research has reported the rs6515831 polymorphism in asthma. Interestingly, some studies on MAVS deserve further attention. It had been reported that MAVS was involved in airway hyperresponsiveness induced by viral infection.^[27]^ In a study on ovarian cancer, eosinophils were significantly negatively correlated with MAVS expression.^[28]^ Therefore, it could be inferred that MAVS might play a role in the pathogenesis of asthma. However, further research is required in this regard.

There was a significant difference in the distribution of NLRP3 rs10925023 genotypes between the asthmatic and healthy groups in this study. Compared with the rs10925023 GG genotype, the rs10925023 TT genotype reduced the risk of asthma. This study was the first to explore the correlation between NLRP3 rs10925023 and asthma. However, previous studies have reported interesting results for other polymorphic loci in NLRP3. Yuki et al found that NLRP3 polymorphisms (rs4612666 and rs10754558) were significantly associated with susceptibility to food induced allergic reactions and aspirin-induced asthma.^[29]^ Another study revealed that the frequency of the CG genotype of NLRP3 rs10754558 was significantly increased in asthmatic patients with low IgE levels.^[30]^ In this study, we analyzed the relationship between IgE levels and gene polymorphisms. Compared with the rs12048215 genotype AA, the level of IgE in patients with the rs12048215 genotype AG was lower. Therefore, the rs12048215 polymorphism may affect IgE levels. In addition, a study found that the C allele of NLRP3 rs4378247 was associated with lower levels of IL-13 production when peripheral blood cells were stimulated with Blomia tropicalis mite crude extract.^[31]^ In summary, NLRP3 polymorphisms affect the risk of asthma. Notably, a previous study on Helicobacter pylori infection in children showed a significant association between the CC genotype of NLRP3 rs10754558 and eosinophils.^[32]^ The effect of NLRP3 polymorphisms on eosinophils counts and IgE levels in asthmatic patients warrants further exploration.

Association studies have shown that multiple genes play a role in asthma susceptibility.^[33]^ Our research explored gene–gene interactions through MDR. The dendrogram model showed that the interaction between rs7272495 and rs10925023 was the strongest and occurred in a synergistic manner. We analyzed and screened 4 haplotypes of NLRP3 rs10925023 and MAVS rs7272495. Among them, two haplotypes (rs10925023T/rs7272495G and rs10925023T/rs7272495A) were statistically significant and confirmed to reduce the risk of asthma. Although the interactions were weak, they supported the hypothesis that the pathogenesis of asthma was affected by multiple genes.

This study had some limitations. First, the sample size of the included subjects was small, which might have biased the results. Second, the clinical data collected were not comprehensive enough, and there was a lack of further analysis of the clinical phenotype of asthma, such as body mass index and therapeutic effect of asthma.

In conclusion, the MAVS rs6515831 and NLRP3 rs10925023 polymorphisms were associated with the risk of asthma in children. Interactions between NLRP3 and MAVS polymorphisms may affect the risk of asthma. The function of NLRP3 and MAVS needs to be confirmed by further research and it is helpful to deeply understand asthma.

## Acknowledgements

We thank the children and their parents for their participation in the study.

## Author contributions

**Conceptualization:** Cai Xulong.

**Data curation:** Cai Xulong, Zhou Li, Yin Tongjin.

**Formal analysis:** Yin Tongjin.

**Funding acquisition:** Cai Xulong.

**Investigation:** Zhou Li, Yin Tongjin.

**Writing – original draft:** Cai Xulong, Zhou Li, Yin Tongjin.

**Writing – review & editing:** Cai Xulong, Zhou Li, Yin Tongjin.
